# EPIGENETIC CLOCKS OF COMPUTED TOMOGRAPHY MEASURES OF FATTY ORGANS

**DOI:** 10.1093/geroni/igz038.2697

**Published:** 2019-11-08

**Authors:** Ake T Lu, Steve Horvath

**Affiliations:** 1 Department of Human Genetics, University of California, Los Angeles, Los Angeles, California, United States; 2 UCLA, Los Angeles, California, United States

## Abstract

DNA methylation (DNAm) based biomarkers collectively known as “epigenetic clock” yielding accurate measure of age in any tissue across the entire life course have associated with a large host of age-related conditions. To study their implications in metabolic syndromes, we analyzed computed tomography (CT) derived measures of adiposity with 1) three widely-used clocks: Horvath, Hannum, and Levine (PhenoAge), and a newly developed clock DNAmGrimAge, and 2) seven DNAm based plasma proteins, the components comprising the DNAmGrimAge. Age-adjusted DNAmGrimAge and several age-adjusted DNAm plasma proteins including plasminogen activation inhibitor 1 (PAI-1) stand our followed by adrenomedullin and leptin. Fatty liver (r ≤ 0.41, P ≤ 2.9E-37) and excess visceral adipose tissue fat (r ≤0.42, P ≤ 1.5E-41) are the most significant CT-based measures of DNAmPAI-1 and DNAmGrimAge. Furthermore, age-adjusted DNAmGrimAge and age-adjusted DNAmPAI-1 continued showing the most significant relationship with lifestyle factors including healthy diet and educational attainment in expected directions.

